# Adaptation Mechanism of Salt Excluders under Saline Conditions and Its Applications

**DOI:** 10.3390/ijms19113668

**Published:** 2018-11-20

**Authors:** Min Chen, Zhen Yang, Jing Liu, Tingting Zhu, Xiaocen Wei, Hai Fan, Baoshan Wang

**Affiliations:** 1 Shandong Provincial Key Laboratory of Plant Stress Research, College of Life Science, Shandong Normal University, Jinan 250014, Shandong, China; m15168849563@163.com (J.L.); m15953147572@163.com (T.Z.); xiaocenwei@163.com (X.W.); biofanhai@163.com (H.F.); 2 Shandong Provincial Key Laboratory of Microbial Engineering, School of Biologic Engineering, Qilu University of Technology (Shandong Academy of Sciences), Jinan 250300, Shandong, China; gina35@126.com

**Keywords:** salt-excluders, molecular mechanism, salt exclusion, soil salinization

## Abstract

Global soil salinization is increasingly a serious threat to agriculture worldwide. Therefore, it is imperative to improve crop salt tolerance as a means of adaptation to saline habitats. Some halophytes and most monocotyledonous crops are salt-excluders. Understanding the regulatory mechanisms of salt exclusion at the molecular level in salt-exclusion plants is critical for improving the salt tolerance of monocotyledonous crops such as maize, wheat, rice, and sorghum. In this review, we summarize recent research into salt-exclusion mechanisms and the genes that underlie them. Findings related to salt exclusion may accelerate the process of breeding tolerant cultivars by using genomic and molecular tools.

## 1. Introduction

Soil salinization is becoming a serious problem worldwide [1,2]. Among 230 million hectares of farmland currently in use globally, 20% are affected by salt and this number increases every year as a result of illogical crop irrigation practices, excessive fertilization, and excessive plowing as well as natural causes such as salt intrusion into coastal zones resulting from the sea-level rise [3,4,5,6]. At present, due to the poor salt tolerance of the crops plants, it is impossible to plant crops on the saline-alkali land. The challenge of feeding about 10 billion people by the next century is forcing agricultural production into salinized wasteland so that it appears likely to be achieved only through a major breakthrough in breeding crops for salinity tolerance [7,8]. Therefore, understanding the salt-tolerant mechanism of plants especially halophytes, which can survive and complete their life cycles in harsh, saline environments, will be a key step in improving crop salt tolerance for adaptation to saline habitats [7].

Halophytes are generally recognized as plants that can survive high concentrations of electrolytes in their environments [9]. Ecologically, halophytes have been defined as “the native flora of saline soils” [10]. In this context, saline soils are those containing solutions with an osmotic pressure (П) of at least 3.3 bar, which is equivalent to 70 mM monovalent salts [calculated from [11,12]. Recently, halophytes refer to the plants that are adapted to saline soil environments and are able to survive and reproduce at salt concentrations of 200 mM or greater [13,14,15]. On the basis of the physiological basis of salt tolerance as well as the accumulation and transport of ions, the German botanist Breckle [16] classified halophytes into three major categories. (A) Recretohalophytes can secrete salt from the plant body to the outside through salt glands (as in *Limonium bicolor*) or into salt bladders for temporary storage and then the salt will scatter from salt bladders when it encounters strong winds or other external stimuli (as in *Chenopodium quinoa*). Salt entering the plant body is secreted by salt glands or salt bladders, which reduces the salt content of plant life tissue and guarantees its normal growth and development in saline soil environments [17,18,19,20,21]. (B) Euhalophytes such as *Suaeda salsa* and *Mesembryanthemum crystallinum*, which are divided into two categories known as leaf succulent euhalophytes and stem succulent euhalophytes accumulate salt in the vacuoles of succulent green tissues of leaves or stems, respectively [14,22,23,24,25,26]. Euhalophytes compartmentalize excessive salt ions, which enter the plant cells into the vacuole. On the one hand, this reduces the water potential of the plant and helps it to absorb water from the saline soil and, on the other hand, this reduces the ions content in cytoplasm and avoids damage to enzymes and biological substances in the cytoplasm [14,22]. (C) Salt-exclusion halophytes, which are also called salt excluders such as reed plant (*Phragmites communis* L.), accumulate much more salt in the vacuoles of parenchyma tissues and parenchyma of roots and xylem than in the shoot [27].

Up to now, many review papers have been published dealing with a salt tolerance mechanism of euhalophytes and recretohalophytes [13,14,20,28,29], and a halophyte database has been established to help exploit the characteristics of halophytes [30]. However, few papers focus on the salt tolerant mechanism of salt excluders.

Salt-exclusion halophytes achieve salt tolerance through salt exclusion [31] by either excluding most of the Na^+^ and Cl^–^ into the soil solution or by accumulating salt ions in the roots and root–stem junctions [32]. Therefore, shoots of these plants can maintain low concentrations of salt and are free from salt damage. A unique feature of salt exclusion in halophytes is that the Na^+^ and Cl^−^ concentrations are significantly lower in shoots than in roots [27]. Under NaCl treatment, the Na^+^ content is significantly increased in both shoots and roots of reed plants, but it is much higher in roots than in shoots [27].

**Figure 1:**
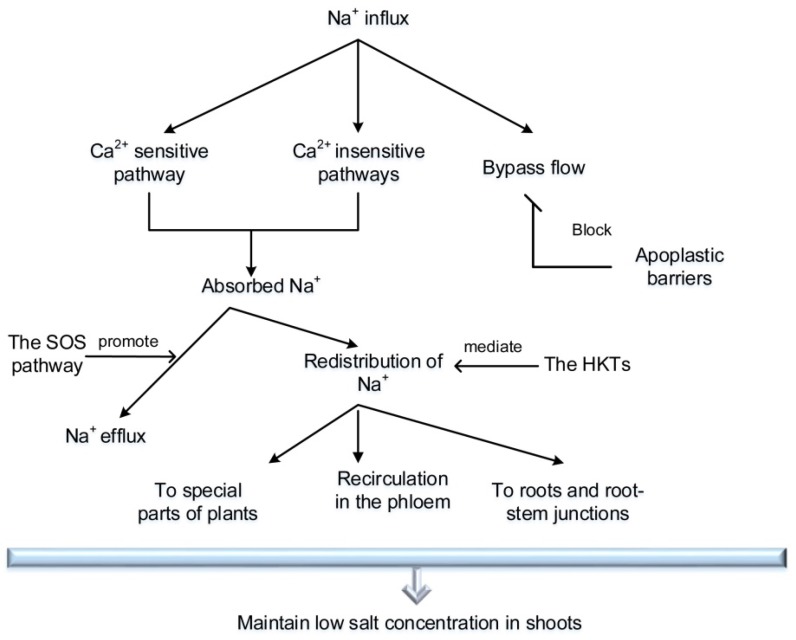
A schematic summary of the sodium-exclusion pathway by which salt-exclusion halophytes and some gramineous plants survive under high-salinity growth conditions. SOS: salt overly sensitive. HKTs: high-affinity potassium transporters.

Most salt-exclusion halophytes only transport 2% of the salt absorbed by the roots to the shoots and the remaining salt is excluded into the soil solution [33]. At present, the key locations for salt exclusion in salt-exclusion halophytes are reported to be the pericycle and xylem parenchyma cells, the root cortex (especially the casparian strip), and the phloem cells [34]. Salt-exclusion halophytes survive in saline habitats by controlling the uptake of Na^+^ and the distribution of Na^+^ (Figure 1).

Most monocotyledon crops have the same salt tolerance mechanism as salt-exclusion halophytes even though monocotyledon crops have a significantly lower salt tolerance than salt-exclusion halophytes. Therefore, understanding the molecular mechanisms of salt tolerance in salt-exclusion plants is very important for improving the salt tolerance of monocotyledon crops such as maize (*Zea mays*), wheat (*Triticum* spp.), rice (*Oryza sativa*), and sorghum. In this review, we summarize the current knowledge about the molecular regulation of salt tolerance in salt-exclusion halophytes and some gramineous plants.

## 2. Control of Na^+^ Uptake

### 2.1. Blocking Na^+^ Influx into the Root

**Figure 2:**
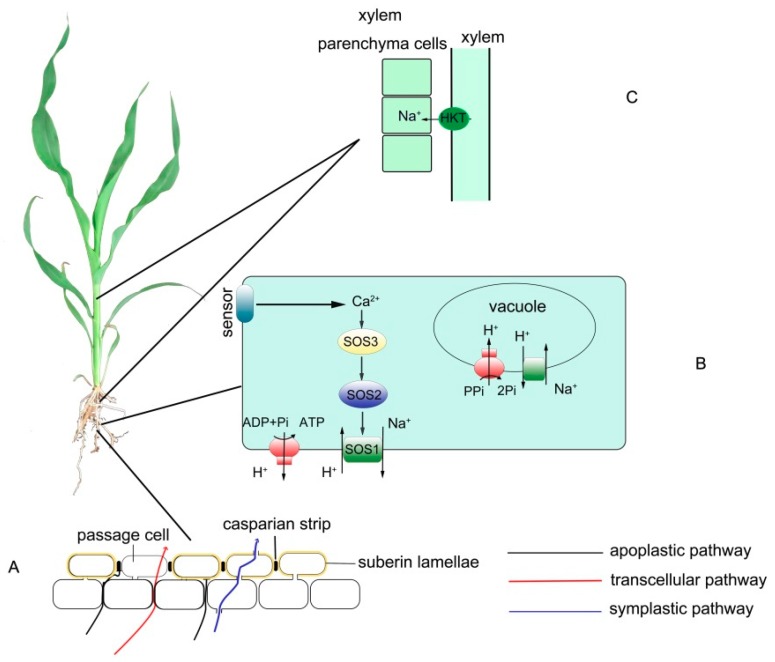
A schematic summary of the key sites and components of salt exclusion in salt-exclusion halophytes and gramineous plants. (**A**) The apoplastic transpiration bypass flow of water and salt ions such as Na^+^ can be blocked by the apoplastic barriers, which consist of casparian bands and suberin lamellae in the endodermis and exodermis of the roots. (**B**) Absorbed Na^+^ can be transported out of the root cell or into the vacuoles of roots and root–stem junction cells by Na^+^/H^+^ antiporters localized in the plasma membrane or the vacuolar membrane. (**C**) HKT1 can mediate the recirculation of Na^+^ back to the roots from the phloem, which can significantly decrease the Na^+^ accumulation in leaves. SOS: salt overly sensitive. HKT: high-affinity potassium transporter.

Na^+^ first enters plants through the epidermis and cortical cells of the root in a passive process known as Na^+^ influx [35]. The pathways of Na^+^ influx consists of Ca^2+^-sensitive and Ca^2+^-insensitive pathways as well as bypass flow [36]. In the Ca^2+^ sensitive pathway, approximately 10 mM Ca^2+^ in the external solution can reduce the Na^+^ influx, which decrease the Na^+^ content in roots and shoots of plants. In this process, it is possible that the SOS (salt overly sensitive) signaling pathway plays an important role and so do non-selective cation channels [1,36]. Two important members of the non-selective cation channels may be the cyclic nucleotide-gate channels (the CNGCs) and the glutamate-activated channels (the GLRs). In the Ca^2+^-insensitive pathway, non-selective cation channels (NSCCs) may be involved and also other possible pathways like HKT1 (high-affinity potassium transporter), KUP (K^+^ uptake permeases), and HAK (high affinity K^+^ transporter) families [32,36]. Among these, it has been shown that bypass flow through apoplastic transpiration plays a major role in the entrance of Na^+^ into shoots [37]. However, apoplastic barriers can block this bypass flow and prevent Na^+^ from entering shoots [38]. Apoplastic barriers consist of the casparian bands (CBs) and suberin lamellae (SLs) in the endodermis and exodermis. CBs can resist the apoplastic water flow while SLs restrict water flow through aquaporins by impeding access to the membranes. In recent years, more attention has been paid to apoplastic barriers since these appear to play important roles in salt exclusion in monocotyledon crops (Figure 2A).

It has been proven that root apoplastic barriers can block the bypass flow of Na^+^ and other solutes entering into the shoots [39]. In addition, previous studies showed that apoplastic barriers play a critical role in controlling salt ion uptake and transport to the shoots in many plants such as maize, wheat, rice, and sorghum [37,40,41]. Once the apoplastic barriers formed, Na^+^, other solutes, and water in the apoplast can reach only the stele and then must be actively transported through channels, carriers, or transporters [42]. Analysis of several rice cultivars found that salt stress induces the strengthening of CBs and SLs in both salt-sensitive and salt-tolerant cultivars. Additionally, salt stress also induced the formation of the CBs and SLs in the endodermis and exodermis close to the root tips. There is a negative correlation between the barrier establishment in roots, on the one hand, and sodium uptake in shoots and plant survival, on the other hand [38,43]. Studies in maize, olive, and mangrove have also revealed the involvement of CBs and SLs in salt stress response [44,45,46,47].

As salt stress can induce the strengthening and development of apoplastic barriers in both the exodermis and the endodermis of roots, scientists wondered whether the amount and monomers of the main components of CBs and SLs change in response to salt stress. According to their experiments, the answer is yes. A doubling of total suberin in the roots of rice was observed after seven days treatment of salt stress. The majority of the salt induced suberin was aliphatics. Among all these newly synthesized aliphatic suberin, ω-hydroxy acids, and fatty acids with chain lengths of C-16, 24, 26, and 28 account for the majority [43]. In addition, growth in stagnant medium markedly increased the total amounts of lignin and aromatic suberin in the roots of rice. As a result, the CBs and SLs were significant enhanced and the NaCl permeability of stagnant roots was markedly decreased. Apoplastic barriers are general structures in plant roots that are present even in the absence of any stress. It has been reported that salt and other abiotic stresses induced the strengthening of apoplastic barriers, which was a relatively slow process that required several days. However, the changes of the expression level of genes related to biosynthesis of components of apoplastic barriers were more forehanded and significant. Notably, salt stress affects the expression of genes related to apoplastic barriers. Lignin, which is synthesized through the phenylpropanoid pathway, is the main component of CBs and several lines of evidence indicated that lignin biosynthesis can be induced by salt stress [48,49]. The biosynthesis of suberin, which is the main component of SLs [50], also seems to be influenced by salt stress. The two main processes of suberin biosynthesis are fatty acid elongation and ω-carbon oxidation [51]. The enzyme β-ketoacyl-CoA synthase (KCS) has been demonstrated to be rate limiting and to determine the chain length of the fatty acid elongation products [52]. Hydroxylation of fatty acids at the ω-position is typically catalyzed by NADPH-dependent cytochrome P450 monooxygenases (P450), which is part of the CYP86 and CYP94 families [53,54,55,56,57]. The expression of the gene encoding P450 (*CYP86A9*) is significantly increased within 30 minutes of the application of salt stress in a salt-tolerant rice cultivar [43]. Expression of *KCS2* and *KCS20* is also enhanced under 100 mM NaCl treatment in *Arabidopsis thaliana* [52]. Conversely, the *horst* (*cyp86a1*) mutant displays a higher hydraulic conductivity as a result of lower suberin content and delayed suberin formation [56]. Moreover, a mutant with ectopic suberization, *esb1*, shows increased Na^+^ accumulation [58,59]. All of these findings support the theory that root apoplastic barriers play a critical role in controlling salt ion uptake and transport to the shoots. 

### 2.2. Enhancing the Na^+^ Efflux in the Root

The net accumulation of Na^+^ in root cortical cells is due to the balance between the influx and efflux of Na^+^. A reduction in the Na^+^ accumulation in the root can be caused by either a decrease in influx or an increase in efflux [36]. Maintaining an ion homeostasis of high K^+^ and low Na^+^ in the cytoplasm is essential for the survival of both halophytes and glycophytes under salt stress. The SOS pathway plays essential roles in the Na^+^ efflux (Figure 2B). Three proteins known as SOS3, SOS2, and SOS1 are involved in the SOS signaling pathway. The SOS3 is a Ca^2+^ binding protein and can be activated by extracellular Ca^2+^. The SOS2 is a protein kinase and can be activated by the SOS3. Then the SOS3 and SOS2 form a complex. The SOS1 is a Na^+^/H^+^ antiporter located on the plasma membrane and can be activated by the complex composed of SOS3 and SOS2. Afterward, it is responsible for efflux of Na^+^ from the cytoplasm under salt stress [60]. Molecular and genetic characterization of several *sos* mutants have shown that the SOS pathway is activated by salt stress and is responsible for reconstruction of Na^+^/K^+^ homeostasis under salt stress [61].

The process by which SOS1 exports Na^+^ from plant cells is an active mechanism requiring energy consumption and is energized by an electrochemical gradient of protons (H^+^) across the plasma membrane (PM). This H^+^ gradient is established by the electrogenic plasma membrane H^+^ pumps (PM H^+^-ATPases), which convert chemical energy derived from hydrolysis of ATP into pH and electrical gradients across the plasma membrane [62,63] so that it constitutes a driving force for the transport of solutes and metabolites across the plasma membrane. The PM H^+^-ATPases are considered to be housekeeping enzymes or dominant enzymes in plants [62,63]. PM H^+^-ATPase activity is controlled by many factors including light, temperature, SOS2-Like Protein Kinase 5 (PKS5) [64], and environmental stresses such as salt stress. Under salt stress, maintaining a high activity of PM H^+^-ATPase is critical for the normal function of SOS1 [22,63,65] 

Under salt stress, due to the increased SOS1 and PM H^+^-ATPase activity in roots, excess Na^+^ in the cytoplasm is either discharged into the extracellular domain of the external solution or discharged into the xylem and then transferred to the shoots.

## 3. Control of Na^+^ Distribution

### 3.1. Reducing Na^+^ Transport to Shoots and Distribution to Roots and Root–Stem Junctions

The salinity tolerance of all plants relies on reduced uptake of Na^+^ and Cl^−^, compartmentalization of Na^+^ and Cl^−^ into special organelles (such as vacuoles), special tissue, or organs (such as roots and root–stem junctions), reduced transport of Na^+^ and Cl^−^ to shoots, and synthesis and accumulation of organic osmotic adjustment substances such as proline, betaine, and soluble sugar [13,66]. Na^+^ in the external solution first enters the epidermis and cortical cells of the plant roots and then enters the root vessels where it is transported to shoots with the xylem sap. During the upward transport, partial Na^+^ is transported out of the xylem by transporters located in the plasma membrane of parenchyma cells around the xylem and then is stored in a salt-insensitive part and the remaining Na^+^ is transported to the shoots with the xylem sap. Although more evidence shows that different transporters are involved in Na^+^ accumulation in different species, the proteins and regulatory networks involved are not clear. To date, there is no credible molecular mechanism to explain apparent differences in Na^+^ absorption rates, in root-to-shoot transport (including xylem loading and phloem recovery), or in the net selectivity of K^+^ or Na^+^ [13,66].

An increasing number of studies have shown that HKT-like transporters are involved in improving plant salt tolerance by increasing the efflux of Na^+^ in the cytoplasm and withdrawing Na^+^ from the xylem sap. In tomato, HKT1;2 can keep the leaf Na^+^/K^+^ ratio low and, therefore, increase salinity tolerance by maintaining Na^+^ homeostasis [67]. In maize, the maintenance of a low Na^+^ concentration in the leaf is essential for salt tolerance. ZmHKT1, which is a maize HKT-type transporter, is preferentially expressed in root stele and it promotes leaf Na^+^ exclusion and increases salt tolerance by withdrawing Na^+^ from the xylem sap [68]. In rice, several members in the *HKT* gene subfamily (*OsHKT1;1*, *OsHKT1;3*, *OsHKT1;4* and *OsHKT1;5*) have been implicated in increased salt tolerance [69].

Even though their expression patterns are different, all HKT members function as cation transporters and especially as Na^+^ transporters. OsHKT1;1, which is located at the plasma membrane, is expressed mainly in the phloem of rice leaf blades and functions in retrieving Na^+^ from the leaf blades [70]. OsHKT1;4 also has a role in Na^+^ exclusion in the leaf sheath [71] at the reproduction stage. OsHKT1;5, which is a Na^+^-selective transporter, is engaged in maintaining a higher K^+^/Na^+^ ratio in shoots. Loss of function of OsHKT1;5 results in massive Na^+^ accumulation in shoots when the rice roots are under salt stress. Salt stress increases OsHKT1;5 transcription in roots and basal stems including basal nodes. Furthermore, immuno-staining assays have shown that OsHKT1;5 is localized in cells adjacent to the xylem in roots, which suggests that it is involved in xylem Na^+^ unloading in leaf sheaths. Moreover, OsHKT1;5 prevents Na^+^ transfer to young leaf blades by mediating Na^+^ exclusion in the phloem. This is a crucial strategy of rice to protect the next generation of seeds and the vital leaf blades under salt stress [3,72,73].

Recent research shows that the rice Mg^2+^ transporter OsMGT1 plays a role in salt tolerance in rice by enhancing OsHKT1;5 activity [74]. Similarly, in bread wheat (*Triticum aestivum* L.), *TaHKT1;5-D*, which encodes a Na^+^-selective transporter, is involved in the maintenance of a higher K^+^/Na^+^ ratio in leaves when the plants are under salt stress. Further research indicates that TaHKT1;5-D is localized on the plasma membrane in the wheat root stele and functions in retrieving Na^+^ from the xylem vessels in the root. Therefore, it plays an essential role in preventing the transport of Na^+^ from the root to the leaves. TaHKT1;5-D may essentially confer salinity tolerance in bread wheat by preventing the transport of Na^+^ from the root to the leaves and increasing shoot Na^+^ exclusion so that the plant can maintain a high K^+^/Na^+^ ratio in the leaves [75].

### 3.2. Na^+^ Distribution to Specific Parts of Shoots 

An important feature of salt tolerance in plants is the ability to limit the transport of Na^+^ to young leaves and growth point under saline conditions while a characteristic of salt-exclusion halophytes and partial gramineous plants is the sequestration of Na^+^ from shoot to old leaves and sheath. In many salt-sensitive species, photosynthetic tissues tend to accumulate high concentrations of Na^+^, which reduce the photosynthetic rate and inhibit plant growth. Our results have shown that salt exclusion is the salt-tolerance mechanism of hybrid Pennisetum (*Pennisetum americanum* × *Pennisetum purpureum*, a biofuel plant). As we increased the NaCl concentration from 0.3% to 0.9%, the Na^+^ content in old leaves and roots increased significantly while the Na^+^ content in functional leaves showed no significant increase. This achieved a certain degree of salt tolerance [76]. A series of transport steps may contribute to Na^+^ uptake in old leaves and sheath. However, to date, it has not been known which candidate genes are involved in the control of these processes. 

A comparison of Na^+^ transport between two varieties of durum wheat (*Triticum turgidum* L. subsp. *durum*) showed that they have different salt tolerances and Na^+^ accumulation abilities. Genetic studies indicated that differences at two major loci related to the regulation of Na^+^ accumulation in the leaf blade led to the phenotypic differences between the two varieties [32]. Additionally, the relatively salt-tolerant cultivar (landrace line 149) shows a high capacity of the leaf sheath to withdraw and accumulate Na^+^ as it enters the leaf while the salt-sensitive cultivar (Tamaroi) does not [77]. Further study uncovered two Na^+^ transporters (Nax1 and Nax2) that control leaf Na^+^ content by restricting the transport of Na^+^ from the root to the shoot and increasing the K^+^/Na^+^ ratio of the leaves. While Nax1 and Nax2 both contribute to limiting the transport of Na^+^ from the root to the shoot, they also show some differences in their salt-response mechanisms. Nax1 transports Na^+^ in leaves to the leaf sheath while Nax2 unloads Na^+^ from the xylem in the root [78]. Further results showed that *Nax1* may be the Na^+^ transporter gene *TmHKT7* [79] and Nax2 may be *HKT1;5* (*HKT8*) [80].

### 3.3. Na^+^ Recirculation in the Phloem

Na^+^ return to the roots by the phloem could significantly decrease Na^+^ accumulation in leaves despite high transport resistance [27]. The pathways for recirculation in the phloem have been reviewed previously [36]. Ren et al. [72] showed that the rice SKC1 (a major quantitative trait locus (QTL) for shoot K^+^ content, to chromosome 1) (OsHKT8) not only regulates the distribution of Na^+^ in the root and shoot but also plays an important role in regulating the recirculation of Na^+^ from the shoot to the root. The expression of the *SKC1* gene is related mainly to the vascular organization of various organs and occurs particularly in the parenchyma surrounding the xylem vessels of nodules, in the internodes, and in the transverse planes of root and sheath base and leaves. It has been speculated that the function of SKC1 in Na^+^ recirculation is to unload Na^+^ from the xylem. Unloading Na^+^ in the xylem of the root can avoid excessive transport of Na^+^ to the shoot while unloading Na^+^ in the xylem of the shoot facilitates Na^+^ recycling (Figure 2C). The return of Na^+^ from the shoot to the root may be beneficial for decreasing harm caused by Na^+^ while the return of Na^+^ to the root may eventually result in Na^+^ excretion through the plasma membrane Na^+^/H^+^ antiporter (SOS1), which eliminates Na^+^ poisoning. In addition, recycling may contribute to an even distribution of Na^+^ throughout the plant, which might permit other mechanisms to eliminate Na^+^ toxicity in order to function more efficiently [72].

## 4. Using Improved Salt Tolerance to Improve Crops

The majority of reports to date related to plant breeding have focused on maintaining or increasing annual yields [81]. However, if new crop cultivates with characteristics of both high-yield and salt-tolerant are bred, they can be used to improve saline-alkali land while improving food production. Therefore, breeding focused on improving the yields of crops under salt stress is becoming increasingly important. Conventional plant breeding has long been used to generate stress-tolerant crop varieties. However, this process is time consuming and labor intensive, relies on the existence of well-characterized germplasms, and can result in the introduction of undesirable traits along with those selected for [82,83]. Therefore, advanced breeding strategies such as marker-assisted selection and genetic engineering seem to be more attractive. It is particularly important to explore the mechanism of salt tolerance in crops, to reveal important pathways in response to salt resistance in crops, and to find key genes related to salt tolerance in crops.

Encouragingly, salt exclusion is found in some form in most crops especially among the salt-exclusion halophytes, which encompass a group that includes most monocotyledon (e.g., grain) species. The key pathways and genes involved in the salt-exclusion processes of different groups of plants are also beginning to be understood, as described above. In addition, the function and mechanism of some key genes related to salt tolerance have also been revealed. This information can be used in combination with QTL, Genome-wide association analyses (GWAS), and genetic engineering techniques to provide guidance for the molecular breeding of crop salt tolerance. After key pathways and genes involved in the salt-exclusion processes are understood, the applications of new technologyies such as CRISPER, RNAi, and overexpression are very important for improving salinity stress tolerance. The overexpression of *SOS1* can reconstruct Na^+^/K^+^ homeostasis of cytoplasm and withdraw more ions, which ensures low ion concentration in the transpiration stream under salt stress [84]. The PKS5 (SOS2-Like Protein Kinase 5) kinase activity can be completely removed or reduced by CRISPR, which is an RNAi technique that can increase the PM H^+^-ATPase activity since PKS5 negatively regulates the PM H^+^-ATPase [64].

Based on the related knowledge, we think that the main aim of future crop breeding are controlling the uptake of Na^+^ and distributing Na^+^ (entering the plant body) to specific parts that are not sensitive to salt. The main directions of breeding are discussed below.

### 4.1. Exploiting the Key Genes Responsible for Forming Apoplastic Barrier (Especially the Casparian Strip)

The apoplastic barrier (especially the casparian strip) plays a critical role in controlling Na^+^ uptake and transport to the shoots. The changes of the expression level of genes related to biosynthesis of components of apoplastic barriers were more forehanded and significant. Our recent research results showed that suberin-forming key genes *(SbKCS11*) of root apoplast barriers in salt exclusion of sweet sorghum plays an important role. It has developed a strategy to resist salinity by accumulating Na^+^ in the roots to limit the excess accumulation of Na^+^ in leaves under salt stress, which plays an important role in the sorghum root rejection of Na^+^ [85]. These factors are responsible for increasing the salt tolerance of sweet sorghum.

### 4.2. Increasing the Activities of the SOS Pathway and the H^+^-ATPase

One of the important functions of the SOS pathway activated by salt stress is to reconstruct Na^+^/K^+^ homeostasis of cytoplasm and exclude excess Na^+^ from root cells under salt stress. Another important function is to maintain the Na^+^ stability in the pericycle where Na^+^ can be transported to the shoot with the transpiration stream. The increased SOS1 and PM H^+^-ATPase activity can withdraw more ions, which will ensure low ion concentration in the transpiration stream. Therefore, the increasing activities of the SOS pathway and the H^+^-ATPase can maintain low Na^+^ concentration in cytoplasmic streams and in the transpiration stream.

### 4.3. Increasing the Activities of HKT-Like Transporters

HKT-like transporters are involved in improving plant salt tolerance by increasing the efflux of Na^+^ in the cytoplasm, withdrawing Na^+^ from the xylem sap, reducing Na^+^ transport to shoots, and distributing Na^+^ to specific parts (These parts are not sensitive to salt such as root, root–stem junctions, leaf sheath, and old leaf). These transporters are responsible for Na^+^ recirculation in the phloem.

## 5. Conclusions

Soil salinity is a severe problem for agriculture worldwide. Exploring the mechanism, pathways, and key genes related to salt tolerance in crops is meaningful for salt-tolerant crops breeding and improvement of saline land. As most monocotyledonous crops are salt-excluders, understanding the mechanisms of salt exclusion at the molecular level is critical for improving the salt tolerance of monocotyledonous crops such as maize, wheat, rice, and sorghum. In the present review, we summarize recent research into salt-exclusion mechanisms and the genes that underlie them. However, even though knowledge on the molecular mechanisms of plant salinity tolerance is dramatically expanding, there remain many uncertainties. Further dissection of the complex regulatory networks of plant salt exclusion tolerance and, in particular, the key genes controlling the apoplastic barrier of plant roots will provide a molecular basis for improving the salt tolerance of crop plants.

## References

[1] Zhu J.K. (2001). Plant salt tolerance. Trends Plant Sci..

[2] Feng Z.T., Deng Y.Q., Zhang S.C., Liang X., Yuan F., Hao J.L., Zhang J.C., Sun S.F., Wang B.S. (2015). K^+^ accumulation in the cytoplasm and nucleus of the salt gland cells of *Limonium bicolor* accompanies increased rates of salt secretion under NaCl treatment using NanoSIMS. Plant Sci..

[3] Deinlein U., Stephan A.B., Horie T., Luo W., Xu G.H., Schroeder J.I. (2014). Plant salt-tolerance mechanisms. Trends Plant Sci..

[4] Han G., Wang M., Yuan F., Sui Na., Song J., Wang B. (2014). The CCCH zinc finger protein gene AtZFP1 improves salt resistance in *Arabidopsis thaliana*. Plant Mol. Biol. Rep..

[5] Smajgl A., Toan T.Q., Nhan D.K., Ward J., Trung N.H., Tri L.Q., Tri V.P.D., Vu P.T. (2015). Responding to rising sea levels in the Mekong Delta. Nat. Clim. Change.

[6] Lin J., Li J.P., Yuan F., Yang Z., Wang B.S., Chen M. (2018). Transcriptome profiling of genes involved in photosynthesis in *Elaeagnus angustifolia L.* under salt stress. Photosynthetica.

[7] Shabala S., Bose J., Hedrich R. (2014). Salt bladders: Do they matter?. Trends Plant Sci..

[8] Yang Z., Wang Y., Wei X., Zhao X., Wang B., Sui N. (2017). Transcription profiles of genes related to hormonal regulations under salt stress in sweet sorghum. Plant Mol. Biol. Rep..

[9] Flowers T.J., Baker D.A., Hall J.L. (1975). Halophytes. Ion Transport in Plant Cell and Tissues.

[10] Jennings D.H. (1976). The effect of sodium chloride on higher plants. Diol. Rev..

[11] Richards L.A. (1954). Diagnosis and improvement of saline and alkali soils. Soil Sci..

[12] Feng Z., Sun Q., Deng Y., Sun S., Zhang J., Wang B. (2014). Study on pathway and characteristics of ion secretion of salt glands of *Limonium bicolor*. Acta Physiol. Plant.

[13] Flowers T.J., Colmer T.D. (2008). Salinity tolerance in halophytes. New Phytol..

[14] Song J., Wang B. (2015). Using euhalophytes to understand salt tolerance and to develop saline agriculture: *Suaeda salsa* as a promising model. Ann. Bot-London.

[15] Guo J., Li Y., Han G., Song J., Wang B.S. (2018). NaCl markedly improved the reproductive capacity of the euhalophyte *Suaeda salsa*. Funct. Plant Biol..

[16] Breckle S.W., Ajmal K.M., Irwin A.U. (1995). How do halophytes overcome salinity. Biology of Salt Tolerant Plants.

[17] Deng Y., Feng Z., Yuan F., Guo J., Suo S., Wang B. (2015). Identification and functional analysis of the autofluorescent substance in Limonium bicolor salt glands. Plant Physiol. Bioch..

[18] Yuan F., Lyu M.A., Leng B.Y., Zheng G.Y., Feng Z.T., Li P.H., Zhu X.G., Wang B.S. (2015). Comparative transcriptome analysis of developmental stages of the *Limonium bicolor* leaf generates insights into salt gland differentiation. Plant Cell Environ..

[19] Yuan F., Lyu M.J., Leng B.Y., Zhu X.G., Wang B.S. (2016). The transcriptome of NaCl-treated Limoniumbicolor leaves reveals the genes controlling salt secretion of salt gland. Plant Mol. Biol. Rep..

[20] Yuan F., Leng B., Wang B. (2016). Progress in studying salt secretion from the salt glands in recretohalophytes: How do plants secrete salt?. Front. Plant Sci..

[21] Kiani-Pouya A., Roessner U., Jayasinghe N.S., Lutz A., Rupasinghe T., Bazihizina N., Bohm J., Alharbi S., Hedrich R., Shabala S. (2017). Epidermal bladder cells confer salinity stress tolerance in the halophyte quinoa and Atriplex species. Plant Cell Environ..

[22] Chen M., Song J., Wang B.S. (2010). NaCl increases the activity of the plasma membrane H^+^-ATPase in C3 halophyte *Suaeda salsa* callus. Acta Physiol. Plant.

[23] Cheng S., Yang Z., Wang M., Song J., Sui N., Fan H. (2014). Salinity improves chilling resistance in *Suaeda salsa*. Acta Physiol. Plant.

[24] Sui N. (2015). Photoinhibition of *Suaeda salsa* to chilling stress is related to energy dissipation and water-water cycle. Photosynthetica.

[25] Chen T., Yuan F., Song J., Wang B. (2016). Nitric oxide participates in waterlogging tolerance through enhanced adventitious root formation in the euhalophyte *Suaeda salsa*. Funct. Plant Biol..

[26] Xu Y., Liu R., Sui N., Shi W., Wang L., Tian C., Song J. (2016). Changes in endogenous hormones and seed-coat phenolics during seed storage of two *Suaeda salsa* populations. Aust. J. Bot..

[27] Matsushita N., Matoh T. (1991). Characterization of Na^+^ exclusion mechanisms of salt-tolerant reed plants in comparison with salt-sensitive rice plants. Physiol. Plant.

[28] Flowers T.J., Galal H.K., Bromham L. (2010). Evolution of halophytes: Multiple origins of salt tolerance in land plants. Funct. Plant Biol..

[29] Flowers T.J., Munns R., Colmer T.D. (2015). Sodium chloride toxicity and the cellular basis of salt tolerance in halophytes. Ann. Bot..

[30] Santos J., Al-Azzawi M., Aronson J., Flowers T.J. (2016). eHALOPH a database of salt-tolerant plants: Helping put halophytes to work. Plant Cell Physiol..

[31] Levitt J. (1980). Responses of Plant to Environmental Stress Chilling, Freezing, and High Temperature Stresses.

[32] Munns R. (2005). Genes and salt tolerance: Bringing them together. New Phytol..

[33] Husain S., Von Caemmerer S., Munns R. (2004). Control of salt transport from roots to shoots of wheat in saline soil. Funct. Plant Biol..

[34] Munns R., James R.A., Läuchli A. (2006). Approaches to increasing the salt tolerance of wheat and other cereals. J. Exp. Bot..

[35] Cheeseman J.M. (1982). Pump-leak sodium fluxes in low salt corn roots. J. Membrane Biol..

[36] Tester M., Davenport R. (2003). Na^+^ Tolerance and Na^+^ Transport in Higher Plants. Ann. Bot-London.

[37] Ochiai K., Matoh T. (2002). Characterization of the Na^+^ delivery from roots to shoots in rice under saline stress: Excessive salt enhances apoplastic transport in rice plants. Soil Sci. Plant Nutr..

[38] Krishnamurthy P., Ranathunge K., Nayak S., Schreiber L., Mathew M. (2011). Root apoplastic barriers block Na^+^ transport to shoots in rice (*Oryza sativa L*.). J. Exp. Bot..

[39] Schreiber L., Zeier J. (1999). Apoplastic barriers in roots: Chemical composition of endodermal and hypodermal cell walls. J. Exp. Bot..

[40] Yeo A., Yeo M., Flowers T. (1987). The contribution of an apoplastic pathway to sodium uptake by rice roots in saline conditions. J. Exp. Bot..

[41] Zhu J.K. (2003). Regulation of ion homeostasis under salt stress. Curr. Opin. Plant Biol..

[42] Barberon M., Geldner N. (2014). Radial transport of nutrients: The plant root as a polarized epithelium. J. Plant Physiol..

[43] Krishnamurthy P., Ranathunge K., Franke R., Prakash H., Schreiber L., Mathew M. (2009). The role of root apoplastic transport barriers in salt tolerance of rice (*Oryza sativa* L.). Planta.

[44] Krishnamurthy P., yothi-Prakash P.A., Qin L., He J., Lin Q., Loh C.S., Kumar P.P. (2014). Role of root hydrophobic barriers in salt exclusion of a mangrove plant Avicennia officinalis. Plant Cell Environ..

[45] Rossi L., Francini A., Minnocci A., Sebastiani L. (2015). Salt stress modifies apoplastic barriers in olive (*Olea europaea L.*): A comparison between a salt-tolerant and a salt-sensitive cultivar. Sci. Hortic-Amsterdam.

[46] Shen J., Xu G., Zheng H.Q. (2015). Apoplastic barrier development and water transport in Zea mays seedling roots under salt and osmotic stresses. Protoplasma.

[47] Tylová E., Pecková E., Blascheová Z., Soukup A. (2017). Casparian bands and suberin lamellae in exodermis of lateral roots: An important trait of roots system response to abiotic stress factors. Ann. Bot-London.

[48] Dixon R.A., Paiva N.L. (1995). Stress-induced phenylpropanoid metabolism. Plant Cell.

[49] Vanholme R., Demedts B., Morreel K., Ralph J., Boerjan W. (2010). Lignin biosynthesis and structure. J. Plant Physiol..

[50] Ranathunge K., Schreiber L., Franke R. (2011). Suberin research in the genomics era—New interest for an old polymer. Plant Sci..

[51] Franke R., Schreiber L. (2007). Suberin-a biopolyester forming apoplastic plant interfaces. Curr. Opin. Plant Biol..

[52] Lee S.B., Jung S.J., Go Y.S., Kim H.U., Kim J.K., Cho H.J., Park O.K., Suh M.C. (2009). Two *Arabidopsis* 3-ketoacyl CoA synthase genes, KCS20 and KCS2/DAISY, are functionally redundant in cuticular wax and root suberin biosynthesis, but differentially controlled by osmotic stress. Plant J..

[53] Benveniste I., Bronner R., Wang Y., Compagnon V., Michler P., Schreiber L., Salaün J.P., Durst F., Pinot F. (2005). CYP94A1, a plant cytochrome P450-catalyzing fatty acid *ω*-hydroxylase, is selectively induced by chemical stress in *Vicia sativa* seedlings. Planta.

[54] Benveniste I., Tijet N., Adas F., Philipps G., Salaün J.P., Durst F. (1998). CYP86A1 from *Arabidopsis thaliana* Encodes a Cytochrome P450-Dependent Fatty Acid Omega-Hydroxylase. BBRC.

[55] Compagnon V., Diehl P., Benveniste I., Meyer D., Schaller H., Schreiber L., Franke R., Pinot F. (2009). CYP86B1 is required for very long chain *ω*-hydroxyacid and *α*, *ω*-dicarboxylic acid synthesis in root and seed suberin polyester. J. Plant Physiol..

[56] Höfer R., Briesen I., Beck M., Pinot F., Schreiber L., Franke R. (2008). The *Arabidopsis* cytochrome P450 CYP86A1 encodes a fatty acid *ω*-hydroxylase involved in suberin monomer biosynthesis. J. Exp. Bot..

[57] Bouquin R.L., Pinot F., Benveniste I., Salaün J.P., Durst F. (1999). Cloning and functional characterization of CYP94A2, a medium chain fatty acid hydroxylase from *Vicia sativa*. Biochem. Biophres. Co..

[58] Baxter I., Hosmani P.S., Rus A., Lahner B., Borevitz J.O., Muthukumar B., Mickelbart M.V., Schreiber L., Franke R.B., Salt D.E. (2009). Root suberin forms an extracellular barrier that affects water relations and mineral nutrition in *Arabidopsis*. PLoS Genet..

[59] Hosmani P.S., Kamiya T., Danku J., Naseer S., Geldner N., Guerinot M.L., Salt D.E. (2013). Dirigent domain-containing protein is part of the machinery required for formation of the lignin-based Casparian strip in the root. Proc. Natl. Acad. Sci. USA.

[60] Liu J., Zhu J.K. (1998). A calcium sensor homolog required for plant salt tolerance. Science.

[61] Shi H., Ishitani M., Kim C.S., Zhu J.K. (2000). The *Arabidopsis thaliana* salt tolerance gene SOS1 encodes a putative Na^+^/H^+^ antiporter. Proc. Natl. Acad. Sci. USA.

[62] Morsomme P., Boutry M. (2000). The plant plasma membrane H^+^ -ATPase: Structure, function and regulation. BBA-Biomembranes.

[63] Palmgren M.G. (2001). Plant plasma membrane H^+^-ATPases: Power-houses for nutrient uptake. Plant Mol. Biol..

[64] Yang Y., Qin Y., Xie C., Zhao F., Zhao J., Liu D., Chen S., Fuglsang A.T., Palmgren M.G., Schumaker K.S. (2010). The *Arabidopsis* Chaperone J3 Regulates the Plasma Membrane H^+^-ATPase through Interaction with the PKS5 Kinase. Plant Cell.

[65] Fuglsang A.T., Guo Y., Cuin T.A., Qiu Q., Song C., Kristiansen K.A., Bych K., Schulz A., Shabala S., Schumaker K.S. (2007). *Arabidopsis* protein kinase PKS5 inhibits the plasma membrane H^+^-ATPase by preventing interaction with 14-3-3 protein. Plant Cell.

[66] Shao Q., Han N., Ding T., Zhou F., Wang B. (2014). SsHKT1;1 is a potassium transporter of the C 3 halophyte *Suaeda salsa* that is involved in salt tolerance. Funct. Plant Biol..

[67] Jaime-Pérez N., Pineda B., García-Sogo B., Atares A., Athman A., Byrt C.S., Olías R., Asins M.J., Gilliham M., Moreno V. (2017). The sodium transporter encoded by the *HKT1*;*2* gene modulates sodium/potassium homeostasis in tomato shoots under salinity. Plant Cell Environ..

[68] Zhang M., Cao Y., Wang Z., Wang Z.Q., Shi J., Liang X., Song W., Chen Q., Lai J., Jiang C. (2017). A Retrotransposon in an HKT1 family sodium transporter causes variation of leaf Na^+^ exclusion and salt tolerance in maize. New Phytol..

[69] Hamamoto S., Horie T., Hauser F., Deinlein U., Schroeder J.I., Uozumi N. (2015). HKT transporters mediate salt stress resistance in plants: From structure and function to the field. Curr. Opin. Biotech..

[70] Wang R., Jing W., Xiao L., Jin Y., Shen L., Zhang W. (2015). The OsHKT1; 1 transporter is involved in salt tolerance and regulated by an MYB-type transcription factor. J. Plant Physiol..

[71] Suzuki K., Yamaji N., Costa A., Okuma E., Kobayashi N.I., Kashiwagi T., Katsuhara M., Wang C., Tanoi K., Murata Y. (2016). OsHKT1;4-mediated Na^+^ transport in stems contributes to Na^+^ exclusion from leaf blades of rice at the reproductive growth stage upon slat stress. BMC Plant Biol..

[72] Ren Z.H., Gao J.P., Li L.G., Cai X.L., Huang W., Chao D.Y., Zhu M.Z., Wang Z.Y., Luan S., Lin H.X. (2005). A rice quantitative trait locus for salt tolerance encodes a sodium transporter. Nat. Genet..

[73] Kobayashi N.I., Yamaji N., Yamamoto H., Okubo K., Ueno H., Costa A., Tanoi K., Matsumura H., Fujii-Kashino M., Horiuchi T. (2017). OsHKT1; 5 mediates Na^+^ exclusion in the vasculature to protect leaf blades and reproductive tissues from salt toxicity in rice. Plant J..

[74] Chen Z.C., Yamaji N., Horie T., Che J., Li J., An G., Ma J.F. (2017). A Magnesium transporter OsMGT1 plays a critical role in salt tolerance in rice. J. Plant Physiol..

[75] Byrt C.S., Xu B., Krishnan M., Lightfoot D.J., Athman A., Jacobs A.K., Watson-Haigh N.S., Plett D., Munns R., Tester M. (2014). The Na^+^ transporter, TaHKT1;5-D, limits shoot Na^+^ accumulation in bread wheat. Plant J..

[76] Wang D., Yuan F., Wang B.S., Chen M. (2012). Response of energy plant hybrid pennisetum to NaCl stress and its salinity threshold. Chinese J. Plant Ecol..

[77] Davenport R., James R.A., Zakrisson-Plogander A., Tester M., Munns R. (2005). Control of sodium transport in durum wheat. Plant Physiol..

[78] James R.A., Davenport R.J., Munns R. (2006). Physiological characterisation of two genes for Na^+^ exclusion in durum wheat: Nax1 and Nax2. Plant Physiol..

[79] Huang S.B., Spielmeyer W., Lagudah E.S., James R.A., Platten J.D., Dennis E.S., Munns R. (2006). A sodium transporter (HKT7) is a candidate for Nax1, a gene for salt tolerance in durum wheat. Plant Physiol..

[80] Byrt C.S., Platten J.D., Spielmeyer W., James R.A., Lagudah E.S., Dennis E.S., Tester M.R. (2007). HKT1; 5-like cation transporters linked to Na^+^ exclusion loci in wheat, Nax2 and Kna1. J. Plant Physiol..

[81] Gilliham M., Able J.A., Roy S.J. (2016). Translating knowledge in abiotic stress tolerance to breeding programs. Plant. J..

[82] Ashraf M. (2010). Inducing drought tolerance in plants: Recent advances. Biotechnol. Adv..

[83] Varshney R.K., Bansal K.C., Aggarwal P.K., Datta S.K., Craufurd P.Q. (2011). Agricultural biotechnology for crop improvement in a variable climate: Hope or hype?. Trends Plant Sci..

[84] Shi H., Lee B., Wu S.J., Zhu J.K. (2003). Overexpression of a plasma membrane Na^+^/H^+^ antiporter gene improves salt tolerance in *Arabidopsis thaliana*. Nat. Biotechnol..

[85] Zhen Y., Xiaocen W., Baoshan W. (2017). Sorghum bicolor (L.) Moench, is a variation of traditional grain sorghum.

